# The Gutenberg health study: associations between occupational and private stress factors and work-privacy conflict

**DOI:** 10.1186/s12889-016-2881-8

**Published:** 2016-02-29

**Authors:** Susan Garthus-Niegel, Janice Hegewald, Andreas Seidler, Matthias Nübling, Christine Espinola-Klein, Falk Liebers, Philipp S. Wild, Ute Latza, Stephan Letzel

**Affiliations:** Institute and Policlinic of Occupational and Social Medicine, Faculty of Medicine, TU Dresden, Fetscherstr. 74, D-01307 Dresden, Germany; Department of Psychosomatics and Health Behaviour, Norwegian Institute of Public Health, Oslo, Norway; FFAW, Freiburg Research Centre for Occupational Sciences, Bertoldstr. 27, D-79098 Freiburg, Germany; Preventive Cardiology and Preventive Medicine, Department of Medicine 2, University Medical Center Mainz, Johannes Gutenberg University Mainz, Langenbeckstr. 1, D-55131 Mainz, Germany; Federal Institute for Occupational Safety and Health (BAuA), Nöldnerstr. 40-42, D-10317 Berlin, Germany; Center for Thrombosis and Hemostasis, University Medical Center Mainz, Langenbeckstr. 1, D-55131 Mainz, Germany; German Center for Cardiovascular Research (DZHK), partner site RhineMain, Mainz, Langenbeckstr. 1, D-55131 Mainz, Germany; Institute of Occupational, Social and Environmental Medicine, University Medical Center, Johannes Gutenberg University Mainz, Obere Zahlbacher Straße 67, D-55131 Mainz, Germany

**Keywords:** Work-privacy conflict, Private life characteristics, Occupational characteristics, Psychosocial working conditions, Gender differences, Gutenberg Health Study

## Abstract

**Background:**

Work-privacy conflict (WPC) is no longer a rarity but constitutes a societal problem. The objectives of the present study were (1) to investigate the distribution and prevalence of WPC among the employed participants in the Gutenberg Health Study at baseline and (2) to study the dependence of WPC on a broad range of private life and occupational characteristics as well as on psychosocial working conditions.

**Methods:**

This analysis is based on a representative, population-based sample of 3,709 employees participating in the Gutenberg Health Study. Descriptive and bivariable analyses were carried out separately for women and men. Distribution and prevalence of WPC were examined according to socio-demographic and occupational characteristics as well as psychosocial working conditions. Further, stepwise selection of Poisson log-linear regression models were performed to determine which socio-demographic and occupational characteristics were most associated with the outcome variable WPC and to obtain adjusted prevalence ratios from the final model. The multivariable analyses were conducted both separately for women and men and with all subjects together in one analysis.

**Results:**

There was a high prevalence of WPC in the present study (27.4 % of the men and 23.0 % of the women reported a high or very high WPC). A variety of factors was associated with WPC, e.g. full-time employment, depression and many of the psychosocial risk factors at work. Also, the multivariable results showed that women were of higher risk for a WPC.

**Conclusions:**

By affecting the individual work life, home life, and the general well-being and health, WPC may lead to detrimental effects in employees, their families, employers, and society as a whole. Therefore, the high prevalence of WPC in our sample should be of concern. Among women, the risk for suffering from WPC was even higher, most likely due to multiple burdens.

**Electronic supplementary material:**

The online version of this article (doi:10.1186/s12889-016-2881-8) contains supplementary material, which is available to authorized users.

## Background

Numerous theories have been used to understand the intersection of work and family life [1]. The spillover theory [2, 3] suggests that experiences in the work domain spill over and affect experiences in the family domain (or vice versa) and has led to a large body of research examining the work-life interface. Generally speaking, work-life interface (or work-life interaction) refers to experiences in the work (family) domain that impact experiences in the family (work) domain [1]. These mutual influences may be both, positive or negative. However, the main research focus has been on the conflicts that may occur between work and family roles [1, 4]. This negative focus may be understood against the background of the changing working conditions in the Western world over the last decades, such as the increase in service industry work [5, 6]. Demands on the employees have also been changing. Flexibility regarding time and location, resiliency and social competency are increasingly becoming key qualifications. Further, the subjective indicator of work intensity, which describes workers’ experience of high demands, reveals an overall increase in work intensity in most European countries over the past two decades [5]. Dual-career couples and single-parent households find themselves struggling to juggle the competing demands of work and their private lives, which may result in an inter-role conflict [7, 8].

Various concepts have been used to describe such inter-role conflict. One common concept is the so-called “work-family conflict” (WFC) or “family-work conflict” (FWC). Also, “work interference with family” (WIF) and “family interference with work” (FIW) have been used synonymously [7]. A valid critique to these terms is that they cover only a segment of the labour force, namely those working women and men who have children or minors living at home. Therefore, it has been suggested to rather use the concept “work-life conflict” in order to also include singles, single parents, dual-income couples without children living at home etc. [9]. “Work-life conflict” however implies that work is not part of the employee’s life, which is naturally not reflecting reality. Therefore, in the current study we choose to apply the term “work-privacy conflict” as we use the COPSOQ scale with the identical name measuring inter-role conflict. This term provides a precise distinction between both life areas and at the same time pertains to the entire private life.

Regarding WPC, two causal directions are conceivable: On the one hand, work-related stress factors such as shift work or precarious working conditions may have a negative impact on family and private life (“work-privacy conflict”). On the other hand, non-occupational factors such as the personal life situation or major life events may conflict with work demands (“privacy-work conflict”). However, the former path appears to be more dominant and here the research evidence is the strongest [9, 10], probably also because the individual employee’s suffering appears to be larger if the private life is affected than vice versa. Consequently, also the present paper relates to that direction.

In a recent meta-analysis, Byron (2005) examined key determinants of a WPC. Particularly work and nonwork variables (opposed to demographic variables such as sex and income) proved to be of importance. Job stress and hours spent at work increased the risk of suffering from a WPC, whereas a flexible schedule had a protective influence. Regarding the nonwork variables, both family stress and family conflict were important antecedents of a WPC [7].

In addition, there are various studies examining health-related effects of WPC [9]. Mental and physical health related outcomes included increased substance abuse (especially problem drinking), greater psychological stress, more frequent depression and other mental disorders, burnout, and other physical symptoms or somatic complaints including lack of appetite, sleep disorders, headaches or fatigue [9, 11–21]. However, results regarding objective health indices such as blood pressure and cholesterol level remain inconclusive [10].

Even though there are already various studies regarding WPC and associated factors, certain research gaps remain to be filled and further primary research has been called for [7, 9, 10]. In general, more European studies are warranted as most studies have been conducted in North America where working conditions are considerably different [7, 22, 23]. More specifically, important work related variables like job level and job type as well as skill level have often not been assessed [7, 10]. In addition, the investigation of private life variables has been limited [10]. For instance, few studies differentiated between caring for children versus adults [7]; and previous research has mostly focused on conflicts between work and family, resulting in selective samples, namely those with employees who have children or minors living at home [9]. Samples in earlier studies have also been selective, because they mainly included middle-to upper-class employees [10].

In the current paper, we therefore examined WPC and a wide-ranging number of variables in a representative population-based sample of employees from the prospective Gutenberg Health Study (GHS). The objectives of the present explorative study were (1) to investigate the distribution and prevalence of WPC among the employed participants in the GHS at baseline and (2) to study the dependence of WPC on a broad range of private life and occupational characteristics as well as on psychosocial working conditions.

## Methods

### Study population

The GHS is designed as a population-based, prospective, single-centre cohort study in the Rhine-Main region in Western Germany [24–26]. The primary aim is to evaluate and improve cardiovascular risk stratification. The GHS sample was drawn randomly from the governmental local registry offices in the city of Mainz and the district of Mainz-Bingen. The sample was stratified 1:1 for sex and residence (urban and rural) and in equal strata for decades of age. Between 2007 and 2012, 15,010 individuals between 35 and 74 years of age were enrolled, and written informed consent was obtained from all participants. Exclusion criteria were insufficient knowledge of the German language and physical or psychological inability to participate in the examinations at the study centre. Ethical approval of the study protocol and sampling design (including the present investigation) was given by the Ethics Commission of the State Chamber of Medicine in Rhineland-Palatinate and by the data protection officer of the University Medical Center of the Johannes Gutenberg University Mainz as well as the Rhineland-Palatinate data protection officer.

For the present study, we excluded subjects older than 64 years of age (*N* = 3,753). In order to analyse an unencumbered sample and to circumvent a “Healthy Worker Effect” we also excluded those with prevalent vascular diseases (i.e. coronary artery disease, myocardial infarction, stroke, peripheral vascular disease; *N* = 346). In addition, 2,911 GHS participants were not eligible because they did not work and 236 participants had to be excluded because of missing data. A randomly selected sample (*N* = 4,055) filled in an alternative questionnaire regarding psychosocial working conditions (which did not contain questions related to WPC). The final sample in this study therefore consisted of 3,709 subjects (with 1,653 women and 2,056 men). There were fewer females (44.6 %) in the sample, as fewer women were employed than men. The mean age of the participants was 48 years (standard deviation (SD) =7.5).

For the current study, a completed STROBE checklist is provided as a supplementary file (see Additional file 1).

### Measures

#### Private life characteristics

Age was measured as *decades of age* (i.e. 35–44, 45–54 and 55–64 years of age). Education was distinguished between *school education* (“certificate of secondary education (9th Grade)”, “general certificate of secondary education (10th Grade)”, “international baccalaureate (12th/13th grade)”, “other certification” and “none”) and *occupational education* (“vocational school/ apprenticeship”, “technical school/master craftsman”, “university of applied sciences”, “other qualification” and “none”). *Socioeconomic status* (*SES*) was measured using a multi-dimensional aggregated index [27]. The dimensions were “school and professional education”, “occupation”, and “income” and the resulting socioeconomic status groups were classified as “low”, “intermediate” and “high”. Marital status was classified as follows in brackets (“married”, “registered partners”, “divorced”, “separated”, “widow(er)” and “single, never married”). Further, the number of *biological children* (“0”, “1–2” and “≥3”), the number of *children below 18 years of age living at home* (“0”, “1–2” and “≥3”) as well as the number of *people living in the household* (“1–2”, “3–4” and “≥5”) were measured. In addition it was assessed how much *time* was *spent caring for children*, *caring for adult relatives*, *time spent on household errands*, *time spent on hobbies* and *time spent on job development* (“0 h/week”, “1–3 h/week” and “≥4 h/week” respectively). Smoking was assessed via both *smoking status* (“never”, “quit 0–2 years ago”, “quit more than 2 years ago” and “current”) and *pack years* (“never smoked”, “<20 pack years”, “20–39 pack years” and “≥40 pack years”). Regarding *alcohol intake* TOAM limits (tolerable upper alcohol intake levels) [28] were used (“no intake”; “intake beneath tolerable limit” i.e., women < =10g/day, men < =20g/day; “intake above tolerable limit” i.e., women >10–40g/day, men >20–60g/day; and “abuse of alcohol” i.e., women >40g/day, men >60g/day). *Depression* was assessed by the Patient Health Questionnaire (PHQ-9) [29] via self-report (PHQ <10 “no depression” vs. PHQ > =10 “depression”).

#### Occupational characteristics

Occupations were manually double-coded according to the classification of occupations of the Federal Statistical Office Germany. The following occupational characteristics were assessed with the respective categories listed in brackets: *form of employment* (part-time vs. full-time employment), *time spent at work* (<40h/week vs. ≥40h/week), *night shift* (yes/no), *amount of night work* (0–6 days/month vs. ≥7days/month), *job complexity level* (“low” (helpers), “medium” (skilled workers), “complex” (specialists) and “very complex” (experts)), *management* (yes/no) and *position* (“worker”, “employee”, “government officials, judges, military employees”, “self-employed/cooperative agriculturalist”, “self-employed in trade, commerce, craftwork, industry, service”, “academic self-employed profession (e.g. physician, attorney, tax consultant)”, “student/trainee” and “caretaker for relatives”).

#### Psychosocial working conditions

The German version of the Copenhagen Psychosocial Questionnaire (COPSOQ) [30] was used to assess psychosocial working conditions. The COPSOQ consists of 5 thematic domains measuring 26 constructs (including WPC). The first four thematic domains represent the psychosocial factors at work: “Demands” (4 scales), “Influence and development” (5 scales), “Interpersonal relations and leadership” (9 scales) and “Further parameters” (1 scale in the present study: “insecurity at work”). The 5th domain represents “Strain” (6 constructs), assessing the reactions of the employees to the workplace situation as internal outcome parameters. The 6 “Strain” scales are “Job satisfaction”, “Intention to leave”, “General health”, “Burnout”, “Cognitive stress” and “Satisfaction with life”.

#### Work-Privacy Conflict (WPC)

Just as with the nature of the construct, there is a lack of consistency with which WPC has been operationalized. Specific problems include the use of single-item measures and adapted and study-developed measures of unknown validity [10]. Allen and colleagues (2000) suggest to either use the WFC measure by Stephens and Sommer (1996), the WFC/FWC measure by Netemeyer (1996), or the WFC measure developed by Carlson, Kacmar, and Williams (1998) [31–33]. However, they favour the last one, as it includes all three forms of inter-role conflict (time-based, strain-based and behaviour-based) and measures both directions of an inter-role conflict [10].

In the present study we decided to use the “work-privacy conflict scale” (WPC scale), which is an established, validated scale and part of the COPSOQ thematic domain “Demands”. It is assessed with 5 items using a 5-point Likert scale, which can be combined and expressed as a percentage. We defined an indication of a WPC as a WPC Score of or exceeding 60 % (this corresponds with the Likert-scale categories indicating high and very high WPC). The 5-item WPC Scale originates from the work-family conflict scale by Netemeyer (1996), but was developed further for the COPSOQ to not only include family but the *entire private life* (changes are indicated in italics) [32]: 1. The demands of my work interfere with my *private and* family life; 2. The amount of time my job takes up makes it difficult to fulfil family *or private* responsibilities; 3. Things I want to do at home do not get done because of the demands my job puts on me; 4. My job produces *stress* that makes it difficult to fulfil family duties; 5. Due to work-related duties, I have to make changes to my plans for *private or* family activities. In all five items the coding of the Likert scale was as follows, 5=”strongly agree", 4=”agree”, 3=”undecided”, 2=”disagree”, 1=”strongly disagree”. The WPC score was obtained by summing all five items and dividing by 5. Reliability was excellent with α = 0.91.

### Statistical analysis

Descriptive analyses were carried out separately for women and men. WPC was categorized as “very low” (<20 %), “low” (20–39 %), “moderate” (40–59 %), “high” (60–79 %) and “very high” (> = 80 %). Also the bivariable analyses were carried out separately for women and men; distribution and prevalence of WPC were examined according to socio-demographic and occupational characteristics as well as psychosocial working conditions. All statistical analyses were performed in R [34]. For the regression analyses, WPC was dichotomized and defined as having a score of > =60 %. Further, stepwise selection of Poisson stepwise log-linear regression models were performed to determine which socio-demographic and occupational characteristics were most associated with the outcome variable WPC and to obtain adjusted prevalence ratios (PR) from the final model. In order to examine the importance of working hours in more detail, we performed an additional sensitivity analysis where we instead of overtime (“Time spent at work (40+ h)”) and part-time employment included working hours per week. The multivariable analyses were conducted both separately for women and men and with all subjects together in one analysis.

For the stepwise selection, the stepAIC function of the MASS Package was used to determine models based on the Akaike information criterion (AIC) starting with a model that included only age (and sex in the combined model) [35].

## Results

### Bivariable results

#### Private life characteristics

More than half of the employed men reported to have a very low WPC (25.0 %) or low WPC (27.0 %) (see Table 1). Especially older men between 55 and 64 years of age had a very low WPC (34.9 %). 7.9 % of all employed men reported to have a very high WPC. Particularly men with a university degree had a high (24.8 %) or very high WPC (10.4 %). Also men with a high SES (9.6 %), those without a hobby (9.3 %) und particularly those who did not spend time on household errands (19.4 %), had a very high WPC. Further, almost a fourth (24.5 %) of all men with depression had a very high WPC. Men who were divorced (10.0 %) or separated (10.5 %) were also more likely to report a very high WPC (Table 1).

**Table 1:** Private life characteristics and WPC among men

			WPC n (%)				
	Mean (SD) of WPC	n	Very low (<20 %)	Low (20–39 %)	Moderate (40–59 %)	High (60–79 %)	Very high (>=80 %)
		2,056	514 (25.0)	555 (27.0)	424 (20.6)	401 (19.5)	162 (7.9)
Age							
35–44	42 (27)	730	146 (20.0)	199 (27.3)	157 (21.5)	160 (21.9)	68 (9.3)
45–54	39 (26)	847	201 (23.7)	236 (27.9)	183 (21.6)	162 (19.1)	65 (7.7)
55–64	34 (27)	479	167 (34.9)	120 (25.1)	84 (17.5)	79 (16.5)	29 (6.1)
Education (school)							
Certificate of Secondary Education (9^th^ Grade)	32 (26)	593	211 (35.6)	159 (26.8)	112 (18.9)	80 (13.5)	31 (5.2)
General Certificate of Secondary Education (10^th^ Grade)	38 (27)	350	97 (27.7)	89 (25.4)	77 (22.0)	59 (16.9)	28 (8.0)
International Baccalaureate (12^th^/13^th^ Grade)	43 (26)	1,100	201 (18.3)	301 (27.4)	233 (21.2)	262 (23.8)	103 (9.4)
Other certification	18 (14)	5	2 (40.0)	3 (60.0)	0 (0.0)	0 (0.0)	0 (0.0)
None	22 (18)	8	3 (37.5)	3 (37.5)	2 (25.0)	0 (0.0)	0 (0.0)
Education (occupational)							
Vocational School/Apprenticeship	33 (26)	724	247 (34.1)	191 (26.4)	147 (20.3)	98 (13.5)	41 (5.7)
Technical School/Master craftsman	38 (25)	360	76 (21.1)	114 (31.7)	81 (22.5)	69 (19.2)	20 (5.6)
University of Applied Sciences	44 (27)	874	166 (19.0)	223 (25.5)	177 (20.3)	217 (24.8)	91 (10.4)
Other qualification	39 (28)	41	10 (24.4)	13 (31.7)	5 (12.2)	10 (24.4)	3 (7.3)
None	40 (28)	52	14 (26.9)	12 (23.1)	12 (23.1)	7 (13.5)	7 (13.5)
SES							
Low (<7.8)	32 (25)	98	34 (34.7)	26 (26.5)	21 (21.4)	14 (14.3)	3 (3.1)
Intermediate (7.8–14)	34 (26)	838	275 (32.8)	227 (27.1)	165 (19.7)	120 (14.3)	51 (6.1)
High (>14)	43 (26)	1,120	205 (18.3)	302 (27.0)	238 (21.3)	267 (23.8)	108 (9.6)
Marital Status							
Married	39 (26)	1,543	381 (24.7)	429 (27.8)	319 (20.7)	295 (19.1)	119 (7.7)
Registered partners	35 (17)	3	0 (0.0)	2 (66.7)	1 (33.3)	0 (0.0)	0 (0.0)
Divorced	38 (28)	140	42 (30.0)	33 (23.6)	27 (19.3)	24 (17.1)	14 (10.0)
Separated	46 (26)	38	6 (15.8)	8 (21.1)	9 (23.7)	11 (28.9)	4 (10.5)
Widow(er)	32 (26)	16	5 (31.3)	6 (37.5)	0 (0.0)	4 (25.0)	1 (6.3)
Single, never married	40 (27)	316	80 (25.3)	77 (24.4)	68 (21.5)	67 (21.2)	24 (7.6)
Biological children							
0	38 (27)	549	150 (27.3)	145 (26.4)	114 (20.8)	98 (17.9)	42 (7.7)
1–2	39 (26)	1,227	295 (24.0)	342 (27.9)	251 (20.5)	241 (19.6)	98 (8.0)
3+	40 (27)	280	69 (24.6)	68 (24.3)	59 (21.1)	62 (22.1)	22 (7.9)
Children < 18y at home							
0	39 (27)	1,932	492 (25.5)	522 (27.0)	395 (20.4)	372 (19.3)	151 (7.8)
1–2-	45 (26)	102	15 (14.7)	28 (27.5)	25 (24.5)	23 (22.5)	11 (10.8)
3+	35 (25)	22	7 (31.8)	5 (22.7)	4 (18.2)	6 (27.3)	0 (0.0)
People in household							
1–2	36 (27)	918	280 (30.5)	233 (25.4)	171 (18.6)	173 (18.8)	61 (6.6)
3–4	42 (26)	972	198 (20.4)	272 (28.0)	217 (22.3)	195 (20.1)	90 (9.3)
5+	40 (26)	154	32 (20.8)	46 (29.9)	34 (22.1)	32 (20.8)	10 (6.5)
Time spent caring for children							
0 h/week	37 (26)	1,355	382 (28.2)	362 (26.7)	269 (19.9)	246 (18.2)	96 (7.1)
1–3 h/week	43 (26)	480	115 (24.0)	17 (3.5)	142 (29.6)	144 (30.0)	62 (12.9)
4+ hours/week	36 (26)	65	16 (24.6)	22 (33.8)	12 (18.5)	11 (16.9)	4 (6.2)
Time spent caring for adult relatives							
0 h/week	39 (27)	1,948	489 (25.1)	530 (27.2)	402 (20.6)	371 (19.0)	156 (8.0)
1–3 h/week	41 (27)	103	24 (23.3)	24 (23.3)	20 (19.4)	29 (28.2)	6 (5.8)
4+ hours/week	44 (25)	4	1 (25.0)	1 (25.0)	1 (25.0)	1 (25.0)	0 (0.0)
Time spent on household errands							
0 h/week	52 (27)	155	20 (12.9)	32 (20.6)	32 (20.6)	41 (26.5)	30 (19.4)
1–3 h/week	38 (26)	1,722	435 (25.3)	482 (28.0)	353 (20.5)	332 (19.3)	120 (7.0)
4+ hours/week	35 (26)	172	57 (33.1)	41 (23.8)	37 (21.5)	25 (14.5)	12 (7.0)
Time spent on hobbies							
0 h/week	44 (27)	429	89 (20.7)	93 (21.7)	92 (21.4)	115 (26.8)	40 (9.3)
1–3 h/week	38 (26)	1,477	374 (25.3)	410 (27.8)	303 (20.5)	275 (18.6)	115 (7.8)
4+ hours/week	30 (23)	146	49 (33.6)	52 (35.6)	29 (19.9)	9 (6.2)	7 (4.8)
Time spent on job development							
0 h/week	38 (27)	1,726	459 (26.6)	463 (26.8)	349 (20.2)	319 (18.5)	136 (7.9)
1–3 h/week	44 (25)	325	55 (16.9)	89 (27.4)	73 (22.5)	82 (25.2)	26 (8.0)
4+ hours/week	35 (12)	4	0 (0.0)	3 (75.0)	1 (25.0)	0 (0.0)	0 (0.0)
Smoking Status							
Never	41 (26)	851	184 (21.6)	230 (27.0)	184 (21.6)	183 (21.5)	70 (8.2)
Quit 0–2 y ago	37 (26)	64	18 (28.1)	12 (18.8)	19 (29.7)	10 (15.6)	5 (7.8)
Quit 2+ y ago	37 (27)	661	191 (28.9)	177 (26.8)	125 (18.9)	120 (18.2)	48 (7.3)
Current	38 (26)	478	121 (25.3)	136 (28.5)	96 (20.1)	86 (18.0)	39 (8.2)
Pack-years (PY)							
Never smoked	41 (26)	851	184 (21.6)	230 (27.0)	184 (21.6)	183 (21.5)	70 (8.2)
<20 PY	37 (26)	871	233 (26.8)	244 (28.0)	174 (20.0)	157 (18.0)	63 (7.2)
20–39 PY	37 (27)	142	42 (29.6)	32 (22.5)	33 (23.2)	24 (16.9)	11 (7.7)
40+ PY	43 (28)	58	10 (17.2)	20 (34.5)	8 (13.8)	13 (22.4)	7 (12.1)
Alcohol intake^a^							
No intake	38 (26)	702	182 (25.9)	187 (26.6)	152 (21.7)	129 (18.4)	52 (7.4)
Intake beneath tolerable limit	40 (26)	759	169 (22.3)	213 (28.1)	168 (22.1)	148 (19.5)	61 (8.0)
Intake above tolerable limit	38 (28)	522	146 (28.0)	132 (25.3)	91 (17.4)	109 (20.9)	44 (8.4)
Abuse of alcohol	39 (27)	71	16 (22.5)	22 (31.0)	13 (18.3)	15 (21.1)	5 (7.0)
Depression							
No	38 (26)	1944	506 (26.0)	536 (27.6)	399 (20.5)	368 (18.9)	135 (6.9)
Yes	58 (26)	110	8 (7.3)	18 (16.4)	24 (21.8)	33 (30.0)	27 (24.5)

^a^TOAM limits for men: beneath tolerable limit < =20g/day; above tolerable limit >20–60g/day; abuse >60g/day

Compared to men, the proportion of employed women who reported a very low WPC (34.0 %) was higher; 23.4 % had a low WPC. Apart from that, distributions were similar to those of the men. Women aged 55–65 years tended to have a very low WPC (38.8 %), whereas a larger proportion of women with a university degree reported high (21.2 %) or very high WPC (9.6 %). Moreover, women with a high SES (11.1 %) und those not spending time on hobbies (11.0 %) more often had a very high WPC. Nearly a fourth (21.6 %) of all women with depression reported to have a very high WPC (Table 2).

**Table 2:** Private life characteristics and WPC among women

			WPC n (%)				
	Mean (SD) of WPC	n	Very low (<20 %)	Low (20–39 %)	Moderate (40–59 %)	High (60–79 %)	Very high (>=80 %)
		1,653	562 (34.0)	387 (23.4)	323 (19.5)	263 (15.9)	118 (7.1)
Alter							
35–44	36 (28)	608	197 (32.4)	148 (24.3)	104 (17.1)	109 (17.9)	50 (8.2)
45–54	35 (27)	689	227 (32.9)	164 (23.8)	148 (21.5)	105 (15.2)	45 (6.5)
55–64	32 (27)	356	138 (38.8)	75 (21.1)	71 (19.9)	49 (13.8)	23 (6.5)
Education (school)							
Certificate of Secondary Education (9th Grade)	30 (28)	367	160 (43.6)	73 (19.9)	63 (17.2)	49 (13.4)	22 (6.0)
General Certificate of Secondary Education (10th Grade)	30 (26)	506	207 (40.9)	117 (23.1)	93 (18.4)	63 (12.5)	26 (5.1)
International Baccalaureate (12th/13th Grade)	40 (27)	760	188 (24.7)	194 (25.5)	163 (21.4)	146 (19.2)	69 (9.1)
Other certification	39 (27)	15	5 (33.3)	3 (20.0)	4 (26.7)	2 (13.3)	1 (6.7)
None	40 (37)	5	2 (40.0)	0 (0.0)	0 (0.0)	3 (60.0)	0 (0.0)
Education (occupational)							
Vocational School/ Apprenticeship	30 (27)	791	332 (42.0)	175 (22.1)	138 (17.4)	103 (13.0)	43 (5.4)
Technical School/Master Craftsman	35 (26)	229	72 (31.4)	56 (24.5)	53 (23.1)	35 (15.3)	13 (5.7)
University of Applied Sciences	41 (27)	534	120 (22.5)	133 (24.9)	117 (21.9)	113 (21.2)	51 (9.6)
Other qualification	39 (27)	33	7 (21.2)	11 (33.3)	8 (24.2)	4 (12.1)	3 (9.1)
None	30 (32)	66	31 (47.0)	12 (18.2)	7 (10.6)	8 (12.1)	8 (12.1)
SES							
Low (<7.8)	25 (27)	61	31 (50.8)	12 (19.7)	10 (16.4)	4 (6.6)	4 (6.6)
Intermediate (7.8–14)	31 (27)	936	379 (40.5)	206 (22.0)	171 (18.3)	124 (13.2)	56 (6.0)
High (>14)	41 (27)	521	152 (29.2)	169 (32.4)	142 (27.3)	135 (25.9)	58 (11.1)
Marital Status							
Married	33 (27)	1,101	391 (35.5)	269 (24.4)	213 (19.3)	154 (14.0)	74 (6.7)
Registered partners	44 (39)	4	1 (25.0)	1 (25.0)	1 (25.0)	0 (0.0)	1 (25.0)
Divorced	36 (29)	181	65 (35.9)	34 (18.8)	36 (19.9)	32 (17.7)	14 (7.7)
Separated	46 (27)	43	7 (16.3)	11 (25.6)	9 (20.9)	10 (23.3)	6 (14.0)
Widow(er)	24 (27)	44	27 (61.4)	4 (9.1)	4 (9.1)	8 (18.2)	1 (2.3)
Single, never married	39 (27)	280	71 (25.4)	68 (24.3)	60 (21.4)	59 (21.1)	22 (7.9)
Biological children							
0	39 (28)	492	137 (27.8)	112 (22.8)	108 (22.0)	86 (17.5)	49 (10.0)
1–2	32 (27)	959	357 (37.2)	235 (24.5)	171 (17.8)	139 (14.5)	57 (5.9)
3+	35 (27)	202	68 (33.7)	40 (19.8)	44 (21.8)	38 (18.8)	12 (5.9)
Children < 18y at home							
0	34 (27)	1,578	535 (33.9)	374 (23.7)	307 (19.5)	250 (15.8)	112 (7.1)
1–2	37 (26)	66	24 (36.4)	10 (15.2)	14 (21.2)	12 (18.2)	6 (9.1)
3+	27 (21)	9	3 (33.3)	3 (33.3)	2 (22.2)	1 (11.1)	0 (0.0)
People in household							
1–2	35 (28)	910	308 (33.8)	203 (22.3)	180 (19.8)	142 (15.6)	77 (8.5)
3–4	33 (26)	637	223 (35.0)	165 (25.9)	115 (18.1)	99 (15.5)	35 (5.5)
5+	37 (26)	90	27 (30.0)	17 (18.9)	24 (26.7)	18 (20.0)	4 (4.4)
Time spent caring for children							
0 h/week	34 (28)	1,109	385 (34.7)	257 (23.2)	221 (19.9)	160 (14.4)	86 (7.8)
1–3 h/week	35 (27)	346	121 (35.0)	77 (22.3)	58 (16.8)	67 (19.4)	23 (6.6)
4+ hours/week	35 (25)	195	56 (28.7)	52 (26.7)	43 (22.1)	36 (18.5)	8 (4.1)
Time spent caring for adult relatives							
0 h/week	34 (27)	1,483	502 (33.9)	357 (24.1)	292 (19.7)	229 (15.4)	103 (6.9)
1–3 h/week	38 (29)	154	54 (35.1)	26 (16.9)	29 (18.8)	31 (20.1)	14 (9.1)
4+ hours/week	34 (32)	14	6 (42.9)	3 (21.4)	1 (7.1)	3 (21.4)	1 (7.1)
Time spent on household errands							
0 h/week	31 (31)	18	8 (44.4)	3 (16.7)	3 (16.7)	3 (16.7)	1 (5.6)
1–3 h/week	36 (27)	1,154	368 (31.9)	286 (24.8)	225 (19.5)	181 (15.7)	94 (8.1)
4+ hours/week	32 (27)	475	185 (38.9)	97 (20.4)	93 (19.6)	78 (16.4)	22 (4.6)
Time spent on hobbies							
0 h/week	41 (29)	300	81 (27.0)	64 (21.3)	55 (18.3)	67 (22.3)	33 (11.0)
1–3 h/week	34 (27)	1,241	429 (34.6)	295 (23.8)	247 (19.9)	188 (15.1)	82 (6.6)
4+ hours/week	25 (24)	108	49 (45.4)	27 (25.0)	21 (19.4)	8 (7.4)	3 (2.8)
Time spent on job development							
0 h/week	34 (27)	1,418	501 (35.3)	321 (22.6)	279 (19.7)	220 (15.5)	97 (6.8)
1–3 h/week	39 (27)	230	59 (25.7)	65 (28.3)	43 (18.7)	43 (18.7)	20 (8.7)
4+ hours/week	18 (10)	3	2 (66.7)	1 (33.3)	0 (0.0)	0 (0.0)	0 (0.0)
Smoking Status							
Never	35 (28)	771	267 (34.6)	186 (24.1)	130 (16.9)	125 (16.2)	63 (8.2)
Quit 0-2y ago	32 (28)	35	11 (31.4)	11 (31.4)	5 (14.3)	6 (17.1)	2 (5.7)
Quit 2 + y ago	35 (27)	463	148 (32.0)	106 (22.9)	114 (24.6)	66 (14.3)	29 (6.3)
Current	34 (27)	383	136 (35.5)	83 (21.7)	74 (19.3)	66 (17.2)	24 (6.3)
Pack-years							
Never smoked	35 (28)	771	267 (34.6)	186 (24.1)	130 (16.9)	125 (16.2)	63 (8.2)
<20 PY	34 (27)	720	243 (33.8)	162 (22.5)	160 (22.2)	111 (15.4)	44 (6.1)
20–39 PY	34 (26)	102	33 (32.4)	25 (24.5)	23 (22.5)	16 (15.7)	5 (4.9)
40+ PY	40 (34)	20	8 (40.0)	2 (10.0)	2 (10.0)	6 (30.0)	2 (10.0)
Alcohol intake^b^							
No intake	34 (27)	810	280 (34.6)	190 (23.5)	158 (19.5)	126 (15.6)	56 (6.9)
Intake beneath tolerable limit	35 (28)	440	152 (34.5)	92 (20.9)	84 (19.1)	76 (17.3)	36 (8.2)
Intake above tolerable limit	34 (26)	370	120 (32.4)	98 (26.5)	73 (19.7)	56 (15.1)	23 (6.2)
Abuse of alcohol	37 (29)	33	10 (30.3)	7 (21.2)	8 (24.2)	5 (15.2)	3 (9.1)
Depression							
No	32 (26)	1,487	541 (36.4)	360 (24.2)	288 (19.4)	215 (14.5)	83 (5.6)
Yes	55 (28)	162	21 (13.0)	25 (15.4)	33 (20.4)	48 (29.6)	35 (21.6)

^b^TOAM limits for women: beneath tolerable limit < =10g/day; above tolerable limit >10–40g/day; abuse >40g/day

#### Occupational characteristics

Regarding occupational characteristics, men who had to work night shifts, had a very high WPC (13.2 %) (Table 3). Also, men with highly complex tasks (10.1 %) and male self-employed academics (12.3 %) often had a very high WPC. Furthermore, men who worked as a manager (11.0 %) and those with full-time employment (8.1 %) frequently had a very high WPC.

**Table 3:** Occupational characteristics and WPC among men

			WPC n(%)				
	Mean (SD) of WPC	n	Very low (<20 %)	Low (20–39 %)	Moderate (40–59 %)	High (60–79 %)	Very high (>=80 %)
Form of Employment							
Full-time	39 (27)	1,990	490 (24.6)	528 (26.5)	417 (21.0)	394 (19.8)	161 (8.1)
Part-time	26 (22)	66	24 (36.4)	27 (40.9)	7 (10.6)	7 (10.6)	1 (1.5)
Time spent at work							
<40 [h/week]	30 (25)	679	241 (35.5)	209 (30.8)	117 (17.2)	79 (11.6)	33 (4.9)
40+ [h/week]	43 (26)	1,312	263 (20.0)	334 (25.5)	288 (22.0)	305 (23.2)	122 (9.3)
Night shift							
No	37 (26)	1,647	437 (26.5)	466 (28.3)	332 (20.2)	304 (18.5)	108 (6.6)
Yes	47 (27)	372	65 (17.5)	78 (21.0)	86 (23.1)	94 (25.3)	49 (13.2)
Night Work							
0–6 [days/month]	38 (26)	1,893	481 (25.4)	519 (27.4)	393 (20.8)	360 (19.0)	140 (7.4)
7+ [days/month]	48 (27)	126	21 (16.7)	25 (19.8)	25 (19.8)	38 (30.2)	17 (13.5)
Job complexity level							
Low	29 (28)	23	10 (43.5)	5 (21.7)	5 (21.7)	1 (4.3)	2 (8.7)
Medium	33 (26)	716	241 (33.7)	195 (27.2)	144 (20.1)	104 (14.5)	32 (4.5)
Complex	40 (27)	488	116 (23.8)	131 (26.8)	93 (19.1)	105 (21.5)	43 (8.8)
Very complex	44 (26)	792	135 (17.0)	213 (26.9)	176 (22.2)	188 (23.7)	80 (10.1)
Management							
No	37 (26)	1,600	448 (28.0)	423 (26.4)	324 (20.3)	293 (18.3)	112 (7.0)
Yes	45 (26)	456	66 (14.5)	132 (28.9)	100 (21.9)	108 (23.7)	50 (11.0)
Position							
Worker	30 (25)	279	104 (37.3)	73 (26.2)	53 (19.0)	37 (13.3)	12 (4.3)
Employee	39 (26)	1,190	286 (24.0)	326 (27.4)	262 (22.0)	216 (18.2)	100 (8.4)
Government officials, judges, military employees	39 (26)	166	39 (23.5)	46 (27.7)	34 (20.5)	37 (22.3)	10 (6.0)
Self-employed/cooperative agriculturalist	47 (25)	43	7 (16.3)	9 (20.9)	10 (23.3)	15 (34.9)	2 (4.7)
Self-employed in trade, commerce, craftwork, industry, service (also freelancers)	43 (27)	312	63 (20.2)	89 (28.5)	54 (17.3)	77 (24.7)	29 (9.3)
Academic self-employed profession (physician, attorney, tax consultant)	45 (29)	65	15 (23.1)	12 (18.5)	11 (16.9)	19 (29.2)	8 (12.3)
Student/trainee	-	-	-	-	-	-	-
Caretaker for relatives	-	-	-	-	-	-	-

Even though some differences were found, distributions were largely the same for women (Table 4). Also women with highly complex tasks (10.3 %) often reported a very high WPC. Moreover, nearly a third of all women in full-time employment had a high (18.8 %) or very high WPC (10.7 %). Just as in men, doing night shifts (16.4 %) and working as manager (12.2 %) was frequently associated with a very high WPC. Similarly to men, 14.0 % of the female self-employed academics reported a very high WPC.

**Table 4:** Occupational characteristics and WPC among women

			WPC n(%)				
	Mean (SD) of WPC	n	Very low (<20 %)	Low (20–39 %)	Moderate (40–59 %)	High (60–79 %)	Very high (>=80 %)
Form of Employment							
Full-time	41 (28)	919	236 (25.7)	203 (22.1)	209 (22.7)	173 (18.8)	98 (10.7)
Part-time	27 (24)	734	326 (44.4)	184 (25.1)	114 (15.5)	90 (12.3)	20 (2.7)
Time spent at work							
<40 [h/week]	31 (27)	1226	468 (38.2)	301 (24.6)	210 (17.1)	183 (14.9)	64 (5.2)
40+ [h/week]	44 (28)	405	89 (22.0)	80 (19.8)	108 (26.7)	76 (18.8)	52 (12.8)
Night shift							
No	33 (27)	1508	542 (35.9)	358 (23.7)	283 (18.8)	228 (15.1)	97 (6.4)
Yes	51 (26)	128	15 (11.7)	24 (18.8)	38 (29.7)	30 (23.4)	21 (16.4)
Night Work							
0–6 [days/month]	34 (27)	1603	554 (34.6)	374 (23.3)	314 (19.6)	251 (15.7)	110 (6.9)
7+ [days/month]	54 (27)	33	3 (9.1)	8 (24.2)	7 (21.2)	7 (21.2)	8 (24.2)
Job complexity level							
Low	27 (30)	82	41 (50.0)	16 (19.5)	10 (12.2)	8 (9.8)	7 (8.5)
Medium	30 (26)	861	357 (41.5)	200 (23.2)	154 (17.9)	103 (12.0)	47 (5.5)
Complex	39 (28)	303	84 (27.7)	65 (21.5)	66 (21.8)	64 (21.1)	24 (7.9)
Very complex	43 (26)	390	75 (19.2)	101 (25.9)	91 (23.3)	83 (21.3)	40 (10.3)
Management							
No	33 (27)	1506	536 (35.6)	358 (23.8)	289 (19.2)	223 (14.8)	100 (6.6)
Yes	47 (27)	147	26 (17.7)	29 (19.7)	34 (23.1)	40 (27.2)	18 (12.2)
Position							
Worker	26 (30)	52	27 (51.9)	10 (19.2)	7 (13.5)	3 (5.8)	5 (9.6)
Employee	33 (27)	1254	450 (35.9)	294 (23.4)	235 (18.7)	195 (15.6)	80 (6.4)
Government officials, judges, military employees	43 (26)	135	25 (18.5)	34 (25.2)	35 (25.9)	29 (21.5)	12 (8.9)
Self-employed/cooperative agriculturalist	49 (26)	10	2 (20.0)	1 (10.0)	2 (20.0)	4 (40.0)	1 (10.0)
Self-employed in trade, commerce, craftwork, industry, service (also freelancers)	37 (28)	149	45 (30.2)	36 (24.2)	30 (20.1)	25 (16.8)	13 (8.7)
Academic self-employed profession (physician, attorney, tax consultant)	43 (28)	43	10 (23.3)	11 (25.6)	10 (23.3)	6 (14.0)	6 (14.0)
Student/trainee	55 (18)	3	0 (0.0)	0 (0.0)	2 (66.7)	1 (33.3)	0 (0.0)
Caretaker for relatives	41 (41)	4	1 (25.0)	1 (25.0)	1 (25.0)	0 (0.0)	1 (25.0)

#### Psychosocial working conditions

Generally, among both women and men, adverse psychosocial working conditions were associated with a higher WPC and favourable psychosocial working conditions with a lower WPC (Tables 5 and 6). In particular, high scores on “quantitative demands“,”emotional demands“, “demands for hiding emotions“, (low)”work ability” and “burnout” were related to a high level of WPC in both women and men. In women, “role conflicts” and “cognitive stress” played an additional important role. On the other hand, “degree of freedom at work”, “quality of leadership”, “social support”, “job satisfaction” and “life satisfaction” were associated with a low WPC in both women and men.

**Table 5:** Psychosocial working conditions as overall mean (SD) and in relation to WPC category, men

		Mean (SD) of COPSOQ Items according to WPC category				
	Overall COPSOQ Mean (SD)	Very low (<20 %)	Low (20–39 %)	Moderate (40–59 %)	High (60–79 %)	Very high (>=80 %)
Quantitative demands (−)	53 (20)	37 (17)	50 (16)	56 (16)	65 (15)	73 (17)
Emotional demands (−)	46 (21)	34 (20)	43 (18)	50 (18)	56 (17)	60 (22)
Demands for hiding emotions (−)	35 (24)	24 (23)	33 (23)	38 (21)	44 (22)	50 (25)
Influence at work (+)	58 (24)	60 (26)	59 (24)	57 (24)	57 (23)	57 (25)
Degree of freedom at work (+)	72 (23)	76 (21)	73 (22)	70 (24)	69 (24)	66 (26)
Possibilities for development (+)	75 (18)	72 (19)	74 (18)	75 (17)	78 (16)	79 (20)
Meaning of work (+)	78 (18)	79 (17)	77 (18)	76 (17)	77 (17)	78 (19)
Workplace commitment (+)	62 (20)	61 (21)	61 (20)	63 (20)	64 (20)	66 (22)
Predictability (+)	65 (22)	67 (21)	66 (21)	64 (21)	63 (23)	64 (24)
Role-clarity (+)	81 (15)	82 (15)	81 (15)	80 (16)	79 (16)	81 (17)
Role-conflicts (−)	41 (19)	35 (20)	39 (17)	43 (18)	46 (17)	50 (17)
Quality of leadership (+)	51 (22)	56 (23)	53 (21)	50 (21)	48 (22)	42 (20)
Social support (+)	65 (19)	70 (19)	65 (18)	64 (18)	61 (18)	57 (19)
Feedback (+)	47 (21)	50 (23)	46 (20)	47 (20)	44 (21)	43 (22)
Social relations (+)	57 (28)	62 (28)	59 (26)	55 (27)	54 (28)	48 (28)
Sense of community (+)	79 (16)	83 (16)	80 (15)	79 (15)	76 (15)	73 (18)
Mobbing (−)	16 (21)	12 (19)	15 (19)	18 (21)	19 (22)	22 (24)
Job insecurity (−)	25 (20)	23 (20)	25 (20)	28 (22)	25 (19)	27 (21)
Job satisfaction (+)	69 (14)	73 (14)	70 (14)	69 (13)	67 (15)	62 (18)
Work ability (−)	12 (20)	6.7 (16)	10 (18)	13 (19)	16 (21)	24 (29)
General health (+)	74 (15)	76 (15)	75 (14)	74 (15)	72 (17)	70 (16)
Burnout (−)	34 (16)	27 (14)	32 (15)	36 (15)	41 (17)	47 (18)
Cognitive stress (−)	24 (18)	19 (17)	22 (16)	25 (17)	28 (18)	31 (21)
Life satisfaction (+)	71 (17)	75 (15)	72 (16)	71 (15)	69 (17)	63 (20)

**Table 6:** Psychosocial working conditions as overall mean (SD) and in relation to WPC category, women

		Mean (SD) of COPSOQ Items according to WPC category				
	Overall COPSOQ Mean (SD)	Very low (<20 %)	Low (20–39 %)	Moderate (40–59 %)	High (60–79 %)	Very high (>=80 %)
Quantitative demands (−)	49 (20)	35 (18)	47 (16)	55 (16)	63 (15)	71 (15)
Emotional demands (−)	47 (23)	32 (22)	46 (20)	54 (19)	59 (20)	69 (18)
Demands for hiding emotions (−)	37 (25)	26 (24)	37 (22)	43 (23)	47 (21)	56 (24)
Influence at work (+)	47 (26)	47 (28)	50 (25)	50 (25)	46 (24)	39 (23)
Degree of freedom at work (+)	62 (26)	68 (24)	61 (27)	61 (25)	56 (29)	49 (27)
Possibilities for development (+)	69 (20)	64 (21)	69 (19)	72 (19)	73 (19)	75 (18)
Meaning of work (+)	76 (18)	76 (19)	76 (17)	75 (18)	76 (18)	74 (19)
Workplace commitment (+)	60 (20)	58 (21)	60 (18)	61 (20)	61 (19)	62 (20)
Predictability (+)	61 (21)	66 (21)	62 (19)	61 (22)	56 (21)	54 (23)
Role-clarity (+)	79 (16)	81 (16)	80 (15)	78 (16)	75 (18)	76 (19)
Role-conflicts (−)	37 (20)	28 (19)	36 (18)	41 (18)	46 (20)	49 (22)
Quality of leadership (+)	52 (23)	58 (24)	55 (22)	48 (23)	46 (22)	41 (23)
Social support (+)	66 (20)	70 (21)	67 (17)	63 (21)	61 (19)	58 (18)
Feedback (+)	42 (21)	44 (23)	44 (20)	43 (22)	39 (19)	36 (21)
Social relations (+)	55 (29)	60 (29)	57 (30)	53 (29)	50 (27)	48 (27)
Sense of community (+)	80 (17)	84 (15)	79 (15)	77 (17)	76 (17)	74 (19)
Mobbing (−)	16 (21)	11 (17)	14 (20)	19 (23)	23 (23)	22 (25)
Job insecurity (−)	23 (20)	21 (19)	24 (20)	23 (20)	26 (21)	28 (23)
Job satisfaction (+)	68 (15)	72 (15)	69 (13)	66 (15)	64 (16)	60 (16)
Work ability (−)	14 (21)	7.8 (17)	11 (17)	17 (22)	19 (23)	32 (30)
General health (+)	73 (17)	76 (16)	74 (16)	71 (18)	70 (19)	65 (18)
Burnout (−)	42 (18)	34 (16)	39 (16)	45 (16)	52 (15)	61 (15)
Cognitive stress (−)	28 (19)	22 (17)	27 (19)	30 (18)	36 (20)	42 (20)
Life satisfaction (+)	70 (19)	74 (19)	73 (16)	67 (20)	66 (18)	59 (19)

Still, some of the favourable psychosocial working conditions, i.e. “possibilities for development” (particularly in women) and “workplace commitment”, were tendentially positively associated with WPC.

### Multivariable results

Table 7 shows the results of the Poisson regression analyses for women and men separately as well as for all subjects together in one analysis. In the regression models, both socio-demographic as well as occupational characteristics were included. Stepwise selection led to somewhat different models for the respective analyses with a little different set of variables (Table 7). For men, the WPC risk (as expressed by the prevalence ratio) was increased when they spent much time at work (more than 40 h a week) and spent time for caring for adult relatives (PR 1.60, 95 % CI 1.29–1.99 and PR 1.24, 95 % CI 1.07–1.43 respectively). Smoking and spending time on household errands was associated with a lower WPC risk (PR 0.72, 95 % CI 0.53–0.97 and PR 0.91, 95 % CI 0.83–0.99 respectively). For women, particularly depression was associated with an elevated risk for a WPC (PR 1.99, 95 % CI 1.53–2.59). Opposed to men, further explanatory variables for an increased WPC risk in women were a high amount of night shifts (PR 1.92, 95 % CI 1.25–2.93), being divorced (PR 1.41, 95 % CI 1.07–1.86) or separated (PR 1.69, 95 % CI 1.06–2.71) and holding a management position (PR 1.35, 95 % CI 1.03–1.76). The regression model including both men and women showed that, contrary to the descriptive results (Tables 1 and 2), in the adjusted multivariable model women had a higher risk for WPC compared to men (Table 7). Further, according to the overall model the WPC risk was reduced for persons with part-time work (PR 0.50, 95 % CI 0.39–0.64), for older persons (PR 0.83, 95 % CI 0.76–0.91) and for persons not spending time on hobbies (PR 0.88, 95 % CI 0.82–0.93). On the other hand, there were a number of variables that were associated with an elevated risk for a WPC: both a higher SES (PR 1.08, 95 % CI 1.06–1.10) and caring for an adult relative (PR 1.15, 95 % CI 1.06–1.24) were related to a WPC. Also depression (PR 1.55, 95 % CI 1.28–1.89) and being separated (PR 1.53, 95 % CI 1.05–2.22) from the partner was associated with an elevated WPC. With regard to occupational characteristics, working night shifts (PR 1.58, 95 % CI 1.33–1.88), time spent at work (PR 1.34, 95 % CI 1.15–1.55) and working as manager (PR 1.22, 95 % CI 1.05–1.42) were important explanatory variables for WPC in the overall model.

**Table 7:** Prevalence ratios for WPC-score >60 %, results of stepwise selection based on Poisson regression model

	PR (95 % CI)		
	Men (*n* = 1,800)	Women (*n* = 1,542)	All (*n* = 3,342)
Sex (Women)			1.25 (1.08–1.44)
Age (PR per 10y increase)	0.83 (0.74–0.93)	0.91 (0.79–1.04)	0.83 (0.76–0.91)
SES	1.08 (1.06–1.11)	1.07 (1.04–1.10)	1.08 (1.06–1.10)
Diabetes (yes)		0.41 (0.12–1.37)	
Smoking (yes)	0.72 (0.53–0.97)		0.80 (0.64–0.99)
Pack−years	1.01 (1.00–1.02)		1.01 (1.00–1.02)
Negative affectivity	1.08 (1.06–1.09)	1.06 (1.04–1.08)	1.07 (1.05–1.08)
Depression		1.99 (1.53–2.59)	1.55 (1.28–1.89)
Biological children (per child)			1.06 (0.99–1.12)
Time spent caring for adult relatives [hours/week]	1.24 (1.07–1.43)		1.15 (1.06–1.24)
Time spent on household errands [hours/week]	0.91 (0.83–0.99)		
Time spent on hobbies [hours/week]	0.90 (0.83–0.97)	0.84 (0.75–0.93)	0.88 (0.82–0.93)
Divorced		1.41 (1.07–1.86)	1.22 (0.98–1.51)
Separated		1.69 (1.06–2.71)	1.53 (1.05–2.22)
Time spent at work (40+ h)	1.60 (1.29–1.99)		1.34 (1.15–1.55)
Night shift	1.73 (1.44–2.07)	1.50 (1.10–2.03)	1.58 (1.33–1.88)
Night Work (7+ days/month)		1.92 (1.25–2.93)	1.33 (1.02–1.72)
Part–time Employment	0.27 (0.07–1.09)	0.48 (0.38−0.62)	0.50 (0.39–0.64)
Management		1.35 (1.03–1.76)	1.22 (1.05–1.42)

Finally, the results of the sensitivity analysis (see Additional file 2) showed that the risk of a WPC was increased by 3 % with each working hour per week (Overall model: PR 1.03, 95 % CI 1.03–1.04; Men: PR 1.03, 95 % CI 1.03–1.04, Women: PR 1.03, 95 % CI 1.02–1.04). Also here, stepwise selection led to somewhat different models with a slightly different set of variables.

## Discussion

With the changing working conditions over the last decades and the resulting psychological stress, WPC is no longer a rarity but constitutes a societal problem [10]. This is reflected in the high prevalence in the present study (27.4 % of the men and 23.0 % of the women reported a high or very high WPC). In a Swiss, nationally representative study from 2009 the prevalence was “merely” 12.5 % [9]. However, in that study, the researchers used another measurement instrument. This instrument comprised only two items and had a rather low reliability (α = 0.53), while our WPC measure had an excellent reliability (α = 0.91). Also, the Swiss study covered the age span 20–64 years, whereas the subjects in our sample were 35–64 years old. Possibly, the younger subjects in the Swiss study had fewer private obligations yet, which might have protected against a WPC.

We found very interesting results regarding sex differences. According to the descriptive results, it seemed like men had a higher risk for having a WPC. The multivariable results however show that after adjusting for a number of confounding variables, women were of higher risk for a WPC. Similar to our descriptive results, Hämmig et al. (2009) also found that men had more often WPC. However, they only conducted their multivariable analyses separately for women and men. Our multivariable results demonstrate that an overall analysis with an adjustment for sex might be essential, as in fact, women seemed to suffer more often from WPC. In line with this, Byron (2005) found that mothers experience more WPC than fathers. Byron hypothesizes that women might tend to take on greater responsibilities for childcare, and therefore experience more distress, when they also have to deal with a considerable workload [7]. That might be why particularly for women being divorced or separated is associated with an increased risk for a WPC. Still, more mothers than fathers keep the children after a separation [36] and being a working single mother is likely to result in role strain [7]. A traditional role allocation can also be seen with regard to household errands. Almost a fifth of all men, who did not spend any time on household errands, reported a very high WPC; and we found a negative association between WPC and time spent on household errands in the multivariable analyses. On the other hand, we found a positive association between WPC and a lot of time spent at work. Among women, such a clear trend was not discernible. The time, they spent on household errands, seemed not to be associated with their WPC and was not selected in the multivariable model for women.

There was a relative strong association between WPC and depression in our study. This is not surprising and has been found previously [37, 38]. On the one hand depression might lead to a negative response bias, so that depressed women would be more likely to report a WPC. On the other hand it is also conceivable that a high WPC (coupled with general fatigue) results in depression [39]. In the multivariable models that were stratified by sex, depression was selected only in the model for women. Hämmig et al. (2009) also found somewhat stronger associations between work-life conflict and mental health impairments among women. Similarly, in a longitudinal study, work-to-family conflict was found to be more detrimental to women's satisfaction and well-being than that of men [40]. An explanation for this difference might be that a negative spillover from work to private life is more stressful for women, because the family role and private life domain is more important to the woman's self-concept and social identity [9].

For both women and men, full-time employment and many of the other psychosocial risk factors at work were associated with an increased WPC. The importance of work strain and the extent of working hours for a WPC are plausible and have been documented before [9, 17, 41]. Härma (2006) therefore argues that the reduction of overtime and long working days would act as a central factor in reducing psychosocial strain and in the prevention of adverse health effects. At the same time we also found some of the favourable psychosocial working conditions (“possibilities for development”, “workplace commitment”) related to WPC, indicating that demanding jobs that interfere with private life also hold positive outcomes for employees.

### Strengths and limitations

In the current study, we examined a representative, population-based sample. Even though the overall GHS study was set up to look at cardio-vascular risk stratification rather than understanding occupational risk distributions, we investigated a broad variety of factors potentially associated with a WPC. In fact, we included considerably more work variables and assessed more extensively private characteristics than most of the previous studies on WPC [7, 9, 10]. Moreover, we considered the entire private life and did not restrict the assessment to family life.

The cross-sectional design constitutes the main limitation of this study. Even though theoretical frameworks concerning the temporal sequence have been suggested [10, 30], we do not know for sure, whether the related factors are a cause or consequence of WPC. Currently, the GHS is assessing 5-year follow-up data. These data will give the opportunity to prospectively analyse WPC and thus provide a better basis for assessing causality.

## Conclusions

By affecting the individual work life, home life, and the general well-being and health, WPC may lead to detrimental effects in employees, their families, employers, and society as a whole [10, 42]. Therefore, the high prevalence of WPC in our sample should be of concern. Among women, the risk for suffering from WPC was even higher, most likely due to multiple burdens. In Germany, many women consider it to be difficult to combine work and private life, which is inter alia reflected in the comparatively low work participation rate among German mothers [43]. In our view, this poses a gender equality problem and calls for (political) solutions.

## Additional files

Additional file 1:
**STROBE checklist.** (DOCX 42 kb) Additional file 2: Table S1. Sensitivity analysis of Poisson regression model with working hours per week instead of overtime and part-time employment. (DOCX 16 kb)
