# Supplementary material for: The Gutenberg health study: associations between occupational and private stress factors and work-privacy conflict
Source: BMC Public Health. 2016 Feb 29;16:192. doi: 10.1186/s12889-016-2881-8 (PMC4853857; doi:10.1186/s12889-016-2881-8)
Supplement: Additional file 2: Table S1. — Sensitivity analysis of Poisson regression model with working hours per week instead of overtime and part-time employment. (DOCX 16 kb) [file 12889_2016_2881_MOESM2_ESM.docx]

**Table S1.** Sensitivity analysis of Poisson regression model (see Table 4) with working hours per week instead of overtime and part-time employment; prevalence ratios are estimated for WPC-score >60%.

|  | **PR (95% CI)** | | |
| --- | --- | --- | --- |
|  | **Men (n=1,800)** | **Women (n=1,542)** | **All (n=3,342)** |
|  |  |  |  |
| **Sex (Women)** |  |  | 1.17 (1.02-1.34) |
| **Age (PR per 10y increase)** | 0.79 (0.71-0.89) | 0.86 (0.75-0.99) | 0.82 (0.75-0.90) |
| **SES** | 1.09 (1.06-1.11) | 1.08 (1.05-1.11) | 1.08 (1.06-1.10) |
| **Dyslipidemia (yes)** | 0.85 (0.71-1.01) |  |  |
| **Smoking (yes)** | 0.68 (0.50-0.92) |  | 0.78 (0.63-0.97) |
| **Pack-years** | 1.01 (1.00-1.02) |  | 1.01 (1.00-1.02) |
| **Negative affectivity** | 1.08 (1.06-1.10) | 1.06 (1.04-1.08) | 1.07 (1.05-1.08) |
| **Depression** |  | 1.96 (1.50-2.56) | 1.54 (1.27-1.87) |
| **Time spent caring for adult relatives [hours/week]** | 1.26 (1.09-1.46) | 1.12 (1.01-1.25) | 1.17 (1.08-1.27) |
| **Time spent on hobbies [hours/week]** | 0.92 (0.84-0.99) | 0.85 (0.77-0.94) | 0.90 (0.84-0.96) |
| **Divorced** |  | 1.43 (1.07-1.90) |  |
| **Separated** |  | 1.88 (1.20-2.94) | 1.54 (1.06-2.24) |
| **Time spent at work (h)** | 1.03 (1.03-1.04) | 1.03 (1.02-1.04) | 1.03 (1.03-1.04) |
| **Night shift** | 1.55 (1.28-1.87) | 1.41 (1.03-1.93) | 1.57 (1.35-1.83) |
| **Night Work (7+ days/month)** |  | 1.67 (1.05-2.66) |  |
| **Management** |  |  | 1.16 (0.99-1.35) |
